# Endovascular Treatment of Large or Giant Basilar Artery Aneurysms Using the Pipeline Embolization Device: Complications and Outcomes

**DOI:** 10.3389/fneur.2022.843839

**Published:** 2022-03-02

**Authors:** Huijian Ge, Xiheng Chen, Kai Liu, Yang Zhao, Longhui Zhang, Peng Liu, Yuhua Jiang, Hongwei He, Ming Lv, Youxiang Li

**Affiliations:** ^1^Beijing Neurosurgical Institute, Capital Medical University, Beijing, China; ^2^Department of Interventional Neuroradiology, Beijing Tian Tan Hospital, Capital Medical University, Beijing, China; ^3^Beijing Engineering Research Center for Interventional Neuroradiology, Beijing, China; ^4^Department of Neurorehabilitation, Capital Medical University School of Rehabilitation Medicine, China Rehabilitation Research Center, Beijing, China; ^5^Department of Neurosurgery, Peking University International Hospital, Peking University, Beijing, China

**Keywords:** basilar artery, aneurysm, pipeline embolization device, large, giant

## Abstract

**Background:**

This study aimed to investigate clinical and angiographic outcomes of Pipeline embolization device (PED) treatment of large or giant basilar artery (BA) aneurysms and examine associated factors.

**Methods:**

Clinical and angiographic data of 29 patients (18 men, 11 women) with large or giant BA aneurysms were retrospectively examined. Mean age was 44.1 ± 21.2 years (range, 30–68). Mean aneurysm size was 22.2 ± 8.3 mm (range, 12.0–40.1).

**Results:**

Mean angiographic follow-up was 18.3 ± 3.4 months (range, 4.5–60). The rate of adequate aneurysmal occlusion (O'Kelly–Marotta grade C–D) was 87%. The overall complication rate was 44.8%; most complications (84.6%) occurred in the periprocedural period. Univariable comparison of patients who did and did not develop complications showed significant differences in aneurysm size (*p* < 0.01), intra-aneurysmal thrombus (*p* = 0.03), and mean number of PEDs used (*p* = 0.02). Aneurysm size (odds ratio, 1.4; *p* = 0.04) was an independent risk factor for periprocedural complications in multivariable analysis. Mean clinical follow-up was 23.5 ± 3.2 months (range, 0.1–65). Nine patients (31%) had a poor clinical outcome (modified Rankin scale score ≥3) at last follow-up, including 7 patients who died. Univariable comparisons between patients with favorable and unfavorable clinical outcomes showed that aneurysm size (*p* = 0.009) and intra-aneurysmal thrombus (*p* = 0.04) significantly differed between the groups. Multivariable analysis showed that aneurysm size (odds ratio, 1.1; *p* = 0.04) was an independent risk factor for poor clinical outcome.

**Conclusion:**

PED treatment of large or giant BA aneurysms is effective and can achieve a satisfactory long-term occlusion rate. However, the treatment complications are not negligible. Aneurysm size is the strongest predictor of perioperative complications and poor clinical outcome.

## Introduction

Large (≥10 mm) or giant (>25 mm) basilar artery (BA) aneurysms have a particularly poor natural history. Frequently, they are clinically characterized by thrombosis or mass effect on the brainstem, which can cause death if left untreated (1, 2). Patients presenting with symptoms related to brainstem compression have a 5.9% annual risk of stroke and a 40% 5-year mortality (3). However, elective treatment of posterior circulation aneurysms using either surgical or traditional endovascular techniques can result in poor outcomes (4).

Conventional endovascular treatment for vertebrobasilar aneurysms is associated with a high recurrence rate and inadequate parent vascular remodeling (5). In the treatment of large or giant aneurysms, Pipeline embolization device (PED; Medtronic, Minneapolis, MN, USA) treatment has a much higher success rate than other endovascular techniques. In addition, the PED enables treatment of fusiform or complex aneurysms that were previously considered untreatable (6, 7). As experience with flow diverters (FDs) has increased, off-label use of the PED for treatment of posterior circulation aneurysms has become more common (7–9); however, safety and efficacy data for PED treatment of large or giant BA aneurysms are lacking. This study describes our experience using the PED to treat these aneurysms, reports our clinical outcomes, and examines factors that affect periprocedural complications and clinical outcomes.

## Materials and Methods

### Study Population

We retrospectively collected the data of consecutive patients with large or giant BA aneurysms who were electively treated using the PED at our center from January 2016 to October 2020. Patient demographics, symptoms at presentation, aneurysm location, specific interventions, and immediate and follow-up clinical and angiographic outcomes were recorded. Aneurysm location on the BA was classified according to segment (proximal or distal) based on the origin of the anterior inferior cerebellar artery. Aneurysmal morphology was classified as saccular or fusiform. No vertebrobasilar dolichoectasia (VBD) were included in our study. All patients provided written informed consent and were informed that use of the PED to treat large or giant BA aneurysms was considered off-label.

### Endovascular Treatment

All patients received dual antiplatelet therapy (clopidogrel 75 mg/d and aspirin 100 mg/d) for at least 5 days before the procedure. Thromboelastography was used to identify patients with low response to clopidogrel: those with inhibition rate <30% were switched to ticagrelor.

Endovascular procedures were performed by experienced interventionalists. All patients underwent general anesthesia and systemic heparinization (3,000 IU bolus followed by infusion at 1,000 IU/h) for the procedure. A triaxial guide-catheter system using a 6-Fr Cook catheter (Cook Medical, Bloomington, IN, USA), 5-Fr or 6-Fr Navien guiding catheter (Medtronic, Minneapolis, MN, USA), and Marksman microcatheter (Medtronic) was used to deploy the PED. If necessary, another biaxial system was introduced into the contralateral vessels (i.e., vertebral artery) to navigate a microcatheter transporting coils or a balloon. PED device size was selected based on parent vessel measurements obtained on working angle views and three-dimensional angiography. Once the PED reached the position of optimal placement, it was released carefully by withdrawing the Marksman catheter and advancing the delivery wire. We preferred to deploy longer PEDs *in situ* and avoided using the push-pull technique with the microcatheter in the aneurysmal lumen. Bridging with an additional PED was performed if the aneurysmal neck was too broad to be covered entirely by a single stent. For complicated vertebrobasilar junction (VBJ) aneurysms that involved the V4 segments of both vertebral arteries, we sacrificed the distal V4 segment of the nondominant vertebral artery to prevent an inflow jet into the aneurysmal sac. After treatment, we recommend that blood pressure be maintained at the lower limit of normal values during the perioperative period. For patients with long segmental disease, prophylactic administration of tirofiban was usually administered. We routinely used methylprednisolone (80 mg, bid) for 3 days after PED treatment of large or giant basilar aneurysms to prevent delayed rupture and worsening mass effect. Dual antiplatelet therapy was continued for at least 6 months after the procedure. Aspirin monotherapy was continued for life.

### Complications and Outcomes

Migration, insufficient opening (<50%), and foreshortening of the PED were defined as technical complications. If neurological symptoms developed after the procedure, head computed tomography (CT) was performed to exclude hemorrhage and magnetic resonance imaging (MRI) was performed to identify any ischemic event. Periprocedural complications were defined as those that developed within 30 days of the procedure. Angiographic follow-up was generally recommended 3–6 months after treatment, preferably using conventional digital subtraction angiography (DSA). CT angiography (CTA) or magnetic resonance angiography (MRA) was performed in patients who refused conventional angiography. Clinical follow-up data were acquired via outpatient office visits and/or telephone. Clinical outcomes were evaluated using the modified Rankin scale (mRS). Favorable outcome was defined as mRS score ≤ 2; poor outcome was defined as score ≥3. Angiographic outcomes were evaluated using the O'Kelly–Marotta (OKM) scale (10): A, total filling; B, subtotal filling; C, entry remnant; and D, no filling.

### Statistical Analyses

Statistical analyses were performed using SPSS software version 25 (IBM Corp., Armonk, NY, USA). Normally distributed continuous variables are presented as means with standard deviation. Non-normally distributed continuous variables are presented as medians with range. Categorical variables are presented as numbers with frequency. Continuous variables were compared using the two-tailed Student's *t*-test or Mann-Whitney test as appropriate. Categorical variables were compared using the chi-square or Fisher's exact test as appropriate. Variables identified in univariable analysis as potential predictors were included in multivariable logistic regression analysis to determine independent predictors of perioperative complications and clinical outcomes after adjusting for potential confounders. *P* < 0.05 was considered significant.

## Results

Patient and aneurysm characteristics, procedural details, complications, and outcomes are summarized in Table 1.

**Table 1:** Patient and aneurysm characteristics, procedural details, complications, and outcomes.

**No**.	**Age(yrs)/Sex**	**Initial mRS /Presentation**	**Vascular risk factors and multiple aneurysms**	**Aneurysm Location**	**Aneurysm type**	**Largest Aneurysm Size(mm)**	**PED no**.	**Aneurysm involving side branches**	**Adjunct Coling**	**VA Sacrifice**	**Periprocedural complication (days)**	**Long- term adverse events(mos)**	**Last angiographic FU (time/OKM)**	**Last clinical FU (time/mRS)**
1	61,F	0/None	No	Distal	Saccular	15.0	1	No	Yes	No			12 mos/D	24 mos/ 0
2	61,F	1/ME	No	Distal	Saccular	13.0	1	Yes	No	No			12 mos/A2	24 mos/0
3	28,F	1/HA	No	Proximal	Saccular	25.0	1	Yes	Yes	Yes			12 mos/D	22 mos/ 0
4	17,M	2/ME	PICA An	Proximal	Fusiform	23.0	1	Yes	No	No			19 mos/D	24 mos/0
5	72,M	1/ME	PcomA An	Distal	Fusiform	29.0	1	No	No	Yes	WorseningME		6 mos/D	22 mos/1
6	17,F	1/HA	No	Proximal	Fusiform	30.0	3	Yes	No	Yes			8 mos/D	21 mos/0
7	12,M	1/HA	No	Distal	Fusiform	33.0	4	Yes	No	No	IS, DRA, died		None	0.1 mos/6
8	56,M	1/HA	HT	Proximal	Fusiform	26.0	2	Yes	Yes	Yes	IS		8mos/D	31 mos/2
9	68,M	1/ME	HT,SM	Distal	Saccular	12.0	1	No	Yes	No			6 mos/D	25 mos/0
10	56,M	0/None	HT,SM	Distal	Fusiform	18.0	1	No	No	No		IST (5)	5 mos/B	21 mos/4
11	37,F	1/ME	No	Distal	Saccular	34.0	2	No	No	No	IS		6 mos/D	15 mos/ 0
12	34,M	1/HA	SM	Distal	Saccular	20.0	1	Yes	Yes	No			4.5 mos/D	23 mos/ 0
13	69,M	2/ME	No	Proximal	Fusiform	31.4	1	No	No	No	DRA, died		None	0.1 mos/6
14	8,F	1/HA	No	Proximal	Fusiform	26.0	2	Yes	Yes	Yes			12 mos/D	65 mos/0
15	34,F	1/HA	No	Proximal	Fusiform	16.2	2	Yes	Yes	No			14.5 mos/D	65 mos/0
16	61,M	2/ME	No	Distal	Saccular	15.0	1	Yes	Yes	No			3 mos/D	55 mos/2
17	49,F	2/ME	No	Proximal	Saccular	39.0	1	Yes	Yes	Yes	WorseningME	Died (11)	None	11 mos/6
18	61,F	0/None	HT	Proximal	Fusiform	22.0	2	Yes	Yes	Yes	IS	IST (11), died	11 mos/C	11 mos/6
19	8,M	1/ME	No	Proximal	Fusiform	26.0	2	No	Yes	Yes	WorseningME		6 mos/D※	40 mos/0
20	52,M	1/HA	HT,SM	Proximal	Fusiform	22.0	2	Yes	No	No	DRA, died		None	1 mos/6
21	68,M	1/ME	HT,SM	Proximal	Fusiform	26.5	2	Yes	No	No	IS	IST (6)	7 mos/C1	13 mos/3
22	31,M	0/None	SM	Distal	Fusiform	13.0	1	Yes	No	No			8 mos/D	29 mos/0
23	49,F	0/None	HT	Distal	Fusiform	13.0	1	Yes	No	No			15 mos;D	38 mos/0
24	31,M	1/ME	SM	Distal	Fusiform	13.8	1	Yes	No	No			15 mos;D	37 mos/0
25	50,M	0/None	HT	Distal	Fusiform	20.0	1	Yes	No	No		IST (19), died	19 mons;B	19 mos/6
26	76,M	1/ME	SM	Distal	Saccular	12.1	1	No	No	No			6 mos,D	32 mos/0
27	67,F	1/IS	HT	Proximal	Saccular	13.5	1	No	No	No			None	6 mos/0
28	19,M	1/ME	No	Distal	Fusiform	17.0	1	No	No	No			7 mos;C	8 mos/0
29	26,M	0/None	No	Proximal	Saccular	40.1	1	Yes	Yes	No	DRA, died		None	0.1 mos/6

*Adjunct, adjunctive; BA, basilar artery; BT, basilar artery trunk; CN, cranial nerve; DAR, delayed aneurysmal rupture; FU, follow-up; HA, headache; HT, hypertension; IST, in-stent thrombosis; IS, ischemic stroke; ME, mass effect; Mos, months; mRS, modified Rankin Scale; PED, Pipelime embolization device; SM, smoking; VA, vertebral artery; Yrs, years*.

### Patient Characteristics

Twenty-nine patients with a large or giant BA aneurysm were treated using the PED during the study period (18 men, 11 women). All had a single BA aneurysm and two had additional aneurysms in another location. Mean patient age was 44.1 ± 21.2 years (range, 8–76). Before treatment, 20 patients presented in excellent neurological condition (mRS score 0–1) and 4 had mild disability (mRS score 2). The most common presenting symptom was mass effect (13 patients, 44.8%). Nine patients (31.0%) presented with headache. One patient (3.4%) presented with ischemic. Five patients (17.2%) had a history of hypertension and three patients (10.3%) had a history of smoking. Five patients (17.2%) had both a history of hypertension and a history of smoking.

### Aneurysm Characteristics and Procedural Details

Seventeen BA aneurysms were large and 12 were giant. Mean aneurysm size was 22.2 ± 8.3 mm (range, 12.0–40.1). Fusiform aneurysms were more common (18 patients, 62.1%). Fifteen BA aneurysms (51.7%) were classified as distal and 14 as proximal. Eight aneurysms (25.8%) presented with intra-aneurysmal thrombus; two (cases 12 and 16) had been previously coiled and required retreatment because of recanalization.

Forty-one PEDs were implanted in the 29 patients. Mean number of PEDs used per patient was 1.4 ± 0.7 (range, 1–4). A single PED was placed in 20 patients (69.0%) and multiple PEDs were placed in nine (31.0%). Adjunctive coiling or balloon placement was performed in 12 patients (41.4%), including six who underwent contralateral vertebral artery sacrifice. Of the 10 VBJ aneurysm patients, six underwent PED placement along with coiling and vertebral artery sacrifice and four underwent placement of a single PED. The PED covered at least one-third of the BA in all patients. Sufficient PED opening was achieved in all patients.

### Angiographic Outcomes

Twenty-three patients (79%) received angiographic follow-up. Twenty-one patients received DSA follow-up, 1 patient received CTA follow-up, and 1 patient received MRA follow-up. Mean angiographic follow-up was 18.3 ± 3.4 months (range, 4.5–60). Complete occlusion (OKM D) was achieved in 17 aneurysms (74%) and near-complete occlusion (OKM C) in three (13%). Therefore, the rate of adequate occlusion (OKM C–D) was 87%. Incomplete occlusion (OKM B) occurred in three aneurysms (13%). The mean time to complete occlusion overall confirmed by the first imaging was 10.0 ± 1.1 months (range, 4.5–18). The mean time to complete occlusion confirmed by the first imaging was significantly longer in fusiform aneurysms than saccular aneurysms (12.1 ± 3.9 months vs. 7.1 ± 3.5 months; *p* = 0.036). In addition, the mean time to complete occlusion confirmed by the first imaging was significantly longer in aneurysms involving branches than in those not involving branches (12.5 ± 3.6 months vs. 6.4 ± 2.7 months; *p* = 0.018).

### Clinical Outcomes and Complications

**Figure 1:**
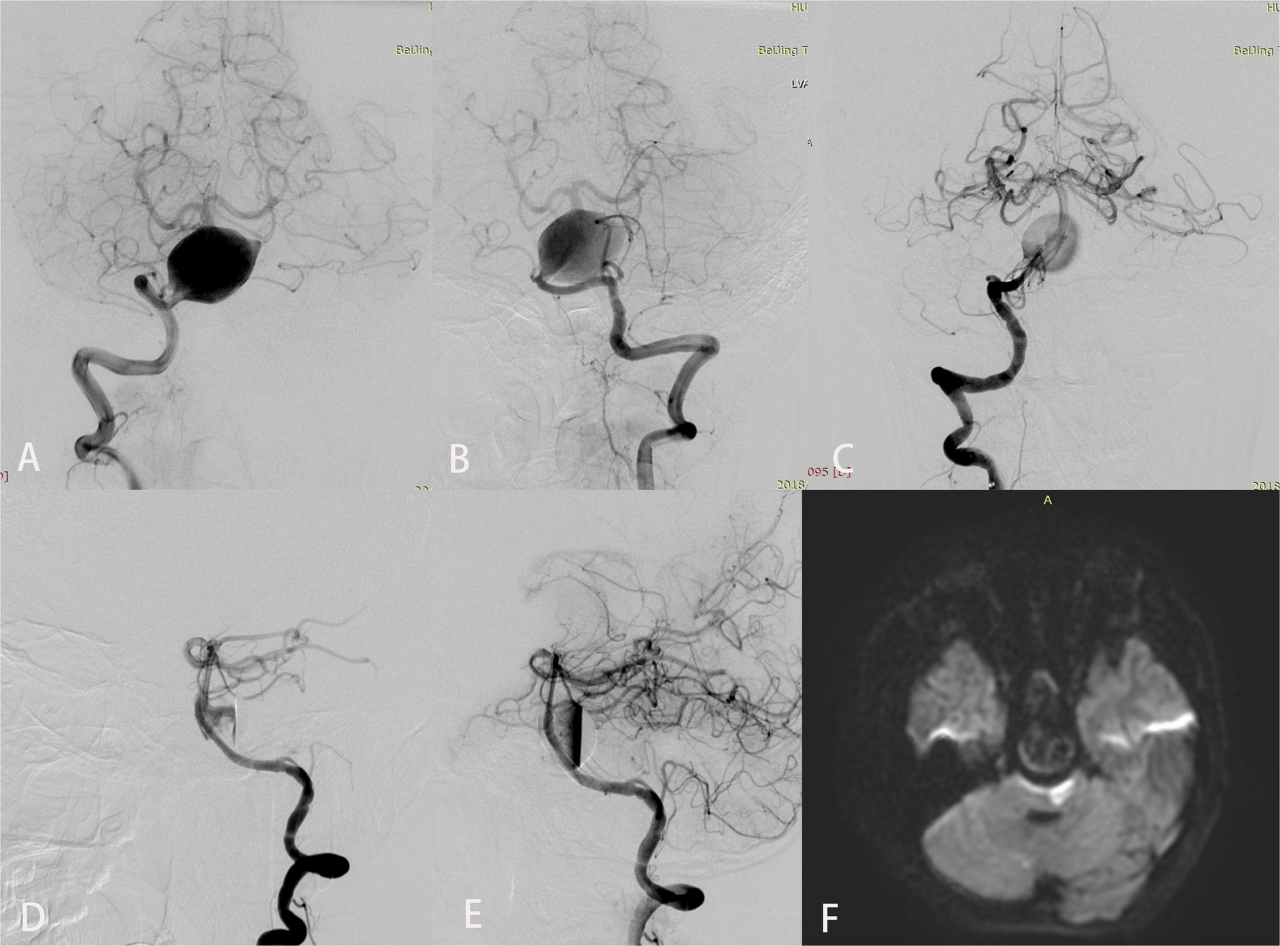
Imaging studies for a 12-year-old boy (case 7) who presented with an 8-month history of chronic headaches and vertigo. Preoperative anteroposterior views of right **(A)** and left **(B)** vertebral angiography showed a giant fusiform basilar artery aneurysm. Anteroposterior **(C)** and lateral **(D)** views of right vertebral angiography immediately after treatment demonstrated excellent reconstruction of the basilar artery with 4 Pipeline embolization devices. An inflow jet is seen in the early arterial phase in **(D)**. **(E)** In the late arterial phase, contrast stasis is seen in the lumen of the aneurysm. Diffusion-weighted imaging **(F)** obtained to evaluate dysarthria and right hemiplegia that developed 12 h after the procedure showed a large brainstem infarct.

Periprocedural complications included ischemic stroke in five patients, worsening mass effect in three, and delayed cerebral hemorrhage in four. Among the ischemic strokes, four were perforator strokes and one was embolic; all five were treated with telescopic PEDs (mean number of PEDs used, 2.4 [range, 2–4]). Case 7 experienced acute onset dysarthria and right hemiplegia 12 h after placement of four PEDs. CT and MRI showed a large brainstem infarct with no hemorrhage. After a 24-h tirofiban infusion, the patient's symptoms gradually resolved. However, on postprocedure day 3, headache and vomiting developed, which rapidly progressed to loss of consciousness, cardiorespiratory arrest, and death before CT could be performed. Relevant imaging studies are shown in Figure 1. Delayed aneurysmal rupture was the presumed cause of death. Other patients who developed neurological symptoms after the procedure also underwent CT to exclude hemorrhage and infusion of tirofiban for 24 h: cases 11 and 18 recovered completely but cases 8 and 21 had mild residual single-limb paresis (final mRS score 2).

**Figure 2:**
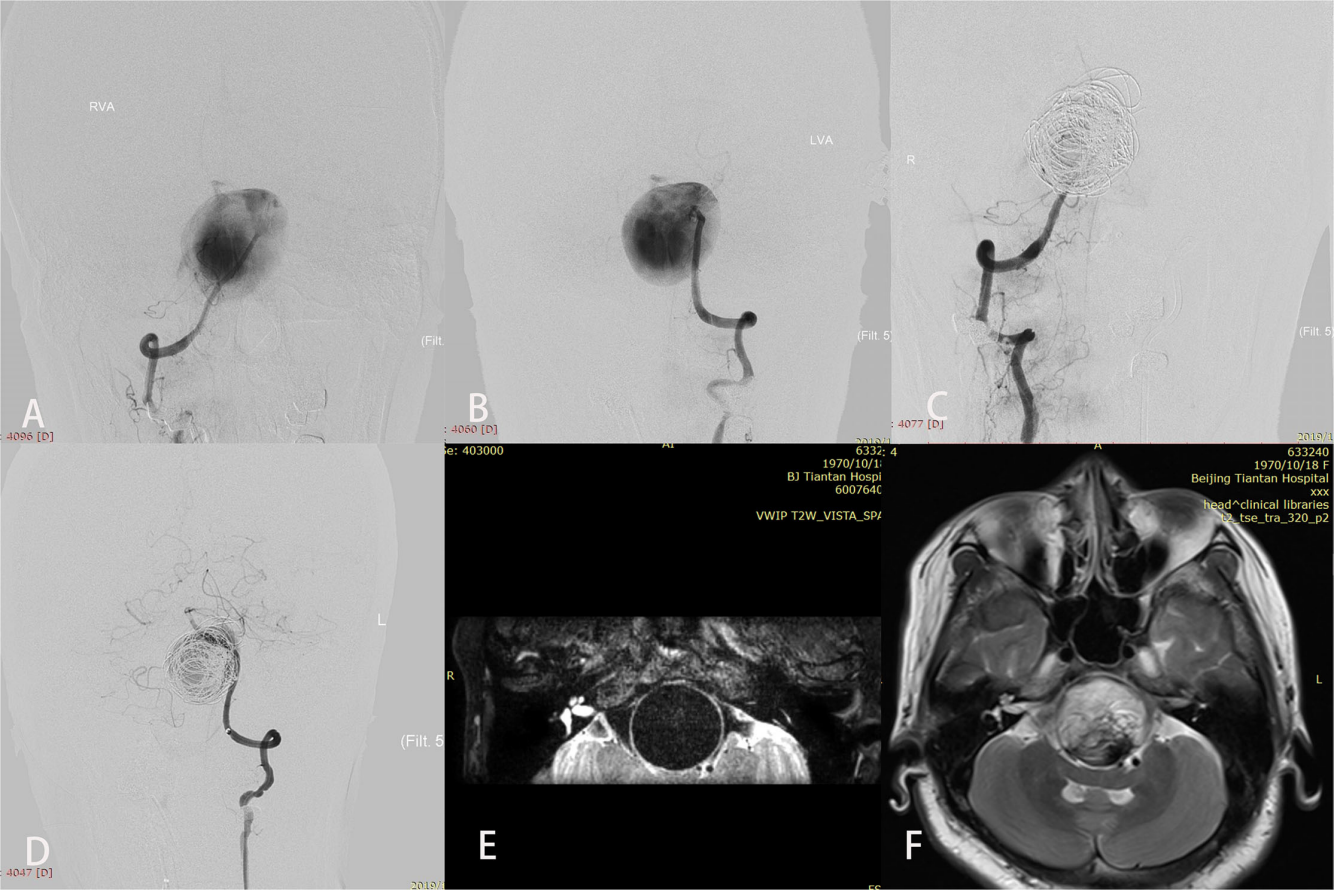
A patient (case 17) with symptoms of mass effect from a giant vertebrobasilar junction aneurysm underwent placement of a single Pipeline embolization device along with coiling and right vertebral artery sacrifice. After treatment, disturbed consciousness and dyspnea developed. Preoperative anteroposterior views of right **(A)** and left **(B)** vertebral angiography showed the aneurysm. **(C,D)** Angiography immediately after the procedure showed successful sacrifice of the right vertebral artery, excellent reconstruction of the basilar artery, and contrast stasis in the lumen of the aneurysm. **(E)** T2-weighted imaging before the intervention demonstrated a complete flow void within the aneurysm. **(F)** T2-weighted imaging after treatment showed aneurysmal enlargement and signal inhomogeneity within the aneurysm consistent with thrombosis.

Three patients with giant aneurysms experienced worsened mass effect after treatment; all presented with initial symptoms of brainstem compression. Cases 5 and 19 experienced abducens nerve palsy after the procedure; however, MRI did not show an infarction. At last follow-up, they had fully recovered. Case 17 developed dyspnea because of aneurysmal brainstem compression after treatment and maintained a tracheotomy until he died of severe pulmonary infection 11 months later (Figure 2).

**Figure 3:**
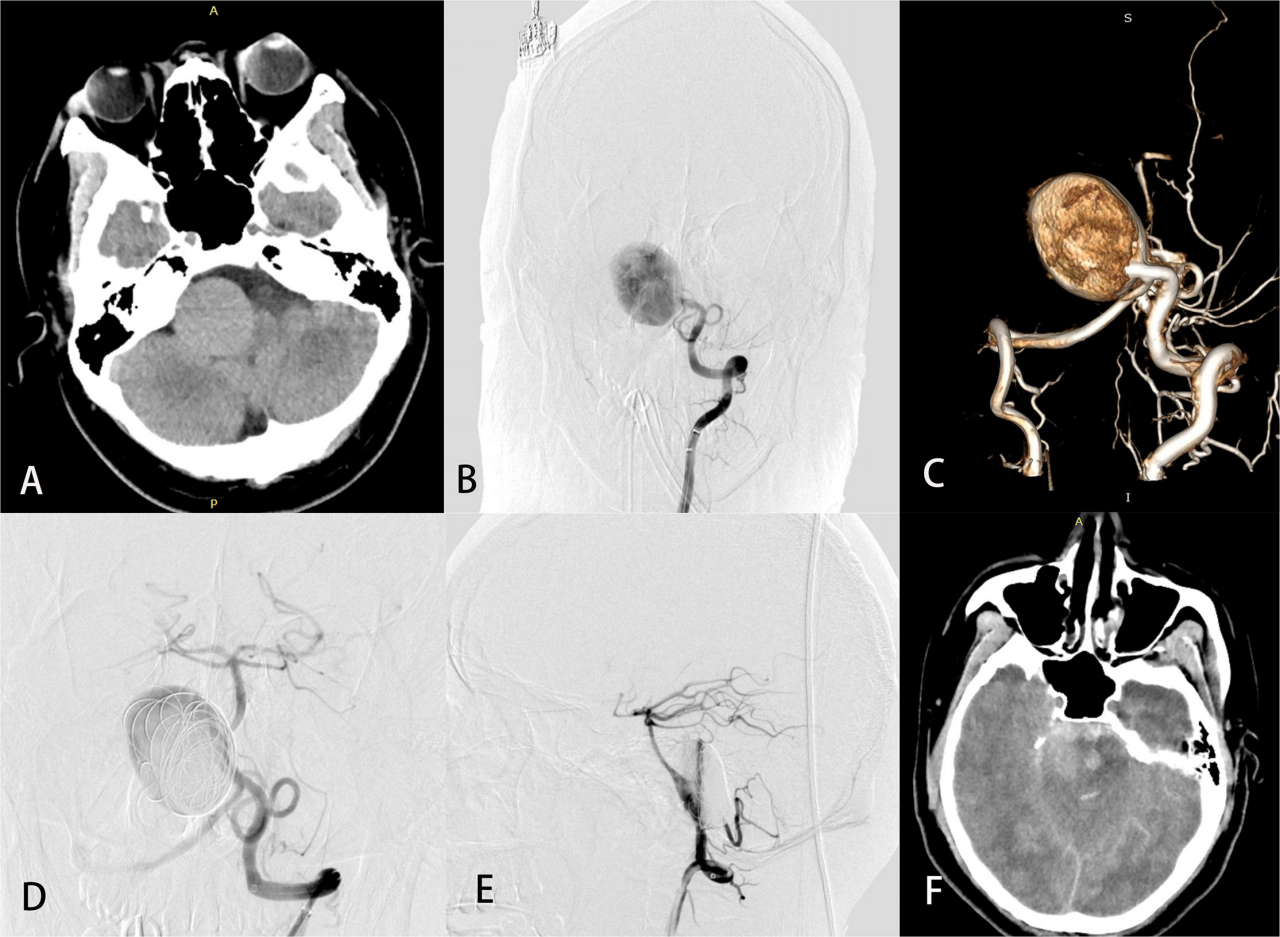
A 26-year-old man (case 29) with a giant basilar artery aneurysm presented with tinnitus. Preoperative computed tomography **(A)** showed a large mass in the right anterior brainstem. Preoperative angiography **(B)** with 3-dimensional reconstruction **(C)** showed a giant side wall saccular aneurysm of the proximal basilar artery. Anteroposterior **(D)** and lateral **(E)** views of left vertebral angiography demonstrated excellent reconstruction of the basilar artery and contrast stasis in the lumen of the aneurysm. Computed tomography **(F)** on postprocedure day 3 was obtained to evaluate headache, vomiting, and disturbed consciousness and revealed massive subarachnoid hemorrhage. The patient later died.

In the perioperative period, four patients with aneurysm size ranging from 22 to 40 mm developed fatal subarachnoid hemorrhage. Three of the four SAHs occurred in patients with aneurysms located on the proximal segment of the BA, including two with a VBJ aneurysm who underwent placement of a single PED without coiling and contralateral vertebral sacrifice. Figure 3 demonstrates an illustrative case (case 29).

Univariable comparison of patients who did and did not develop complications showed significant differences in aneurysm size (30.6 ± 6.3 mm vs. 18.5 ± 6.0 mm; *p* < 0.01), intra-aneurysmal thrombus (54.5 vs. 11.1%; *p* = 0.03), and mean number of PEDs used (1.9 ± 0.9 vs. 1.2 ± 0.5; *p* = 0.02). Aneurysm size (odds ratio, 1.4; 95% confidence interval, 1.0–1.8; p = 0.04) was an independent risk factor for periprocedural complications in multivariable analysis (Table 2).

**Table 2:** Univariable and multivariable analyses of factors associated with perioperative complications.

	**Univariable Analysis**			**Multivariable**
				**Analysis; OR**
				**(95% CI)**,
				***p*-Value**
**Parameter**	**Perioperative**	**No-**	***p*-**	
	**complications**	**perioperative**	**Value**	
	**(*n* = 11)**	**complications**		
		**(*n* = 18)**		
Age	46.4 ± 22.7	42.7 ± 20.8	0.66	
Sex (Male)	8/11 (72.7%)	10/18 (55.6%)	0.45	
Smoking	2/11 (18.2%)	6/18 (33.3%)	0.67	
Hypertension	4/11 (36.4%)	5/18 (27.8%)	0.69	
Initial presention	8/11 (72.7%)	6/18 (33.3%)	0.06	
Intra-aneurysmal thrombus	6/11 (54.5%)	2/18 (11.1%)	0.03	17.1 (0.8–360.4), *p* = 0.07
**Aneurysm location**				
Proximal BA	9/11 (81.8%)	10/18 (55.6%)		
Distal BA	2/11 (18.2%)	8/18 (44.4%)		
Aneurysm involving side branches	7/11 (63.6%)	11/18 (61.1%)	0.60	
Largest aneurysm size (mm)	29.9 ± 6.2	17.5 ± 5.4	<0.01	1.4 (1.0–1.8), *p* = 0.04
**Aneurysm type**				
Fusiform	8/11 (72.7%)	8/18 (44.4%)	0.25	
Saccular	3/11 (27.2%)	10/18 (55.6%)		
Median no. of PEDs (range)	1 (1–3)	2 (2–4)	0.02	2.3 (0.2–23.6), *p* = 0.49
Adjunct Coling	7/11 (63.6%)	5/18 (27.8%)	0.51	

*BA, basilar artery; CI, confidence interval; no., number; OR, odds ratio; PEDs, Pipeline embolization devices*.

In-stent thrombosis occurred in four patients (13.8%) during follow-up. All had a fusiform aneurysm. Cases 10, 21, and 18 developed in-stent thrombosis at 5, 6, and 11 months after the procedure, respectively; clopidogrel was discontinued in all three because of acute gastrointestinal bleeding. In case 25, in-stent thrombosis occurred 19 months after the procedure because antiplatelet therapy was stopped for an orthopedic surgical procedure. Although blood flow through the PED was restored in all four after emergency thrombolytic therapy and subsequent intra-arterial thrombectomy, the patients still experienced severe neurological deficits. Furthermore, angiography after thrombectomy revealed that these aneurysms were not completely occluded. At last follow-up, cases 10 and 21 had mRS scores of 4 and 3, respectively, while cases 18 and 25 died a short time after thrombectomy.

Overall, mean clinical follow-up was 23.5 ± 3.2 months (range, 0.1–65) and the complication rate was 44.8%. Twenty-one patients (72.4%) achieved a favorable clinical outcome (mRS score ≤ 2) or experienced clinical improvement at last follow-up. Nine patients (31%) experienced a poor clinical outcome (mRS score ≥3), including two patients with severe disability and seven patients who died. Overall rates of morbidity and mortality were 10.3 and 24.2%, respectively. The main causes of death were delayed aneurysmal rupture (*n* = 4), in-stent thrombosis (*n* = 2) and worsened mass effect (*n* = 1). Univariable comparisons between patients with favorable and unfavorable clinical outcomes showed that aneurysm size (28.0 ± 8.2 mm vs. 19.6 ± 7.0 mm; *p* = 0.009) and intra-aneurysmal thrombus (55.6 vs. 15%, *p* = 0.04) significantly differed between the groups. Multivariable regression analysis showed that aneurysm size (odds ratio, 1.1; 95% confidence interval, 1.0–1.3; *p* = 0.04) was an independent risk factor for poor clinical outcome (Table 3).

**Table 3:** Univariate and multivariate analyses of factors associated with unfavorable clinical outcome.

	**Univariable Analysis**			**Multivariable**
				**Analysis; OR**
				**(95% CI)**,
				***p*-Value**
**Parameter**	**Favorable**	**Unfavorable**	***p*-**	
	**clinical**	**clinical**	**Value**	
	**outcomes**	**outcomes**		
	**(*n* = 20)**	**(*n* = 9)**		
Age	49.2 ± 18.9	41.8 ± 22.2	0.39	
Sex (Male)	11/20 (55%)	7/9 (77.8%)	0.41	
Smoking	5/20 (25%)	3/9 (33.3%)	0.68	
Hypertension	4/9 (44.4%)	5/9 (55.6%)	0.09	
Initial presention	9/20 (45%)	5/9 (55.6%)	0.70	
Intra-aneurysmal thrombus	3/20 (15%)	5/9 (55.6%)	0.04	5.5 (0.8–40.1), *p* = 0.09
**Aneurysm location**				
Proximal BA	11/20 (55%)	8/9 (88.9%)	0.11	
Distal BA	9/20 (45%)	1/9 (11.1%)		
Aneurysm involving side branches	11/20 (55%)	7/9 (77.8%)	0.41	
Largest aneurysm size (mm)	28.0 ± 8.2	19.6 ± 7.0	0.009	1.1 (1.0–1.3), *p* = 0.04
**Aneurysm type**				
Fusiform	9/20 (45%)	7/9 (77.8%)	0.13	
Saccular	11/20 (55%)	2/9 (22.2%)		
Median no. of PEDs (range)	1 (1–3)	1 (1–4)	0.93	
Adjunct Coling	9/20 (45%)	3/9 (33.3%)	0.69	

*BA, basilar artery; CI, confidence interval; no., number; OR, odds ratio; PEDs, Pipeline embolization devices*.

## Discussion

Large or giant aneurysms involving the BA are less common than those involving the vertebral artery. Despite advances in endovascular and surgical treatment, complex vertebrobasilar artery aneurysms remain difficult to treat (11). In a series of 21 surgically treated patients, Nakatomi et al. (2) reported early postoperative morbidity and mortality rates of 47.6 and 14.3%, respectively; at last follow-up, the respective rates were 71.4 and 57.1%. In another study of 19 patients with large or giant BA aneurysms who were treated with stenting or stent-assisted coiling, Mu et al. (5) reported postoperative complications or poor neurologic outcome in five (26.3%); overall mortality was 15.8% and complete occlusion was achieved in only 20% at last angiographic follow-up.

The PED is another treatment option in patients with complex posterior circulation aneurysms. Use of the PED can achieve better outcomes than surgical or other endovascular techniques (6, 7). To our knowledge, our study is the largest one to date that has examined PED treatment of large or giant BA aneurysms. Our angiographic results are encouraging, as 87% of aneurysms achieved adequate occlusion (OKM C–D). Table 4 summarizes the findings of 10 previous studies comprising five or more patients that reported FD treatment of large or giant BA aneurysms. When pooling these studies' data, the calculated rate of complete occlusion is 75.6%, which is in line with our complete occlusion rate (74%) and far superior to rates achieved by conventional endovascular treatment (5, 22).

**Table 4:** Summary of large series (>5 patients) reporting flow diverter treatment of basilar artery aneurysms.

**Reference**	**FD type**	**All BA cases**	**Mean size (mm)**	**No. of FD**	**Complication** **(%)**	**Ischemic complications** **(%)**	**Hemorrhagic complications** **(%)**	**Mass effect** **(%)**	**Morbidity** **(%)**	**Mortality** **(%)**	**CO at FU** **(total FU cases,%)**
Zhou et al. (13)	PED	7	25.4	13	3 (42.9)	2 (28.6)	0 (0)	0 (0)	1 (14.2)	1 (14.2)	5 (6,83.3)
Dmytriw et al. (8)	PED/FRED	16	20.2	16	3 (18.8)	0 (0)	2 (12.5)	1 (6.3)	2 (12.5)	1 (6.3)	11 (14,71.7)
Tascher et al. (14)	Surpass	26	17.7	46	NA	NA	1 (3.8)	NA	NA	8 (30.6)	NA
Da Ros et al. (15)	PED/SILK/FRED	5	20	5	2 (40)	1 (20)	1 (20)	0 (0)	1 (20)	1 (20)	5 (5,100)
Natarajan et al. (17)	PED	8	14.5	14	2 (25)	1 (12.5)	0 (0)	0 (0)	1 (12.5)	0 (0)	8 (8,100)
Monteith et al. (19)	PED	5	26.2	10	2 (40)	1 (20)	1 (20)	0 (0)	1 (20)	1 (20)	1 (4,25)
Toma et al. (20)	PED/SILK	8	>10	NA	5 (62.5)	2 (25)	1 (12.5)	1 (12.5)	2 (25)	3 (37.5)	NA
Siddiqui et al. (12)	PED/SILK	7	20.8	34	5 (71.4)	3 (42.9)	2 (28.6)	0 (0)	1 (14.3)	4 (57.1)	NA
Kulcsar et al. (16)	SILK	12	11.5	12	5 (41.7)	5 (41.7)	0 (0)	0 (0)	3 (25)	0 (0)	7 (12,58.3)
Byrne et al. (21)	SILK	7	>10	NA	3 (37.5)	1 (12.5)	0 (0)	2 (25)	1 (12.5)	2 (25)	NA
Total		101		150/86	30/75	16/75	8/101	4/86	11/86	21/101	37/49
Total (mean)				1.7	30 (40)	16 (21.3)	8 (7.9)	4 (4.6)	11 (12.7)	21 (20.7)	37 (49,75.6)

*BA, basilar artery; CO, complete occlusion; FD, flow diverter; FRED, flow redirection endoluminal device; FU, follow-up; NA, data not available; PED, pipeline embolization device*.

This superiority may be related to our long angiographic follow-up period. Studies have shown that aneurysms treated using the PED are more likely to achieve complete occlusion over time compared with aneurysms treated using conventional endovascular treatment (23, 24). Complete exclusion of an aneurysm from the circulation requires formation of neointima (25), which begins at the site of contact between the FD and the parent artery. For fusiform aneurysms involving the BA, neoendothelialization requires a longer time in arteries with longer segments of disease, as shown in a histopathological study that reported that thrombosis and endothelial coverage of the FD may not occur before 1 year (18, 26). Complete aneurysm occlusion is also limited in aneurysms involving branches. Continued inflow from a side branch may affect the ability of the FD to reduce aneurysmal inflow and may limit the degree of stasis within the aneurysm, which negatively affects the ultimate outcome of treatment (23, 27). Our observations were similar: fusiform aneurysms and aneurysms involving branches took longer to completely occlude compared with saccular and aneurysms that did not involve branches, respectively.

A recent systematic review of posterior circulation aneurysm patients reported a 22% rate of major complications after flow diversion, with VBJ and BA aneurysms having the worst outcomes (28). As shown in Table 4, the pooled complication rate, morbidity, and mortality among 101 patients with large or giant BA aneurysms treated with FDs was 40, 12.7, and 20.7%, respectively; the overall incidence of adverse outcome was 33.4%.

Our overall complication rate (44.8%) and mortality (24.2%) were slightly higher. Most complications (84.6%) occurred perioperatively. Aneurysm size was an independent risk factor for periprocedural complications and poor clinical outcome. Mean aneurysm size in our study was 22.2 ± 8.3 mm, which is larger than the mean size reported in other series and may explain our higher complication rate. Liang et al. (24) suggested that giant posterior circulation aneurysms (>25 mm) are associated with a high incidence of periprocedural complications. Kiyofuji et al. (9) also reported that large or giant posterior circulation aneurysms are associated with an unfavorable safety profile and poor outcome. Our findings are consistent with previous studies.

Large, partially thrombosed aneurysms in the posterior circulation are prone to thrombus propagation, which can lead to perforator infarction or in-stent thrombosis (29, 30). The most common complication in our study was ischemia, including four perioperative perforator strokes, one perioperative embolic stroke, and four cases of in-stent thrombosis owing to discontinuation of antiplatelet therapy. This result was also similar to a previously reported meta-analysis on FD treatment of posterior circulation non-saccular aneurysms (31). Circumferential involvement of the vessel wall and presence of vital perforating arteries are frequently encountered problems when treating large or giant BA aneurysms. Moreover, the larger the aneurysm and the longer the segment of the BA involved, the more perforator arteries that are damaged. Lesions that involve a great length require the deployment of multiple devices to reconstruct the parent artery. Placement of multiple overlapping PEDs may increase resistance to perforator artery filling caused by the increased surface coverage area (7), which further compounds the perforator injury. Multiple PEDs may also cover more perforators. In our study, perioperative ischemic stroke was associated with greater aneurysm size and the use of multiple PEDs. Siddiqui et al. (12) reported two brain stem ischemic events in seven patients with large or giant fusiform BA aneurysms treated using FDs; the mean number of stents placed per patient was 4.8. Lopes et al. (11) indicated that use of ≥3 PEDs is a strong predictor of major neurological morbidity and mortality. Phillips et al. (32) reported three perforator strokes that occurred in patients treated using a single PED partially or entirely within the BA, which indicates that coverage of a perforating artery ostium may not be the only etiology of stroke in this subset of patients. Heterogeneity in aneurysm size and type between the studies may explain the discrepancy. Although use of multiple PEDs was not an independent risk factor for periprocedural complications in our multivariable analysis, we preferentially deployed a single longer and larger PED *in situ* and avoided using the push/pull technique with the microcatheter in the lumen of aneurysm to reduce perforator coverage and intra-aneurysmal thrombus propagation. We also administered tirofiban prophylactically within 24 h of embolization in patients with long segmental lesions who underwent placement of multiple PEDs, which may explain the absence of serious neurological complications in our patients who experienced perioperative ischemia.

Another more troublesome ischemic complication in our study was in-stent thrombosis. Four patients developed in-stent thrombosis at 5, 6, 11, and 19 months after treatment, respectively. Two experienced severe neurological deficits and two died. Klisch et al. (33) reported two patients with large fusiform basilar trunk aneurysms who developed in-stent thrombosis after clopidogrel was stopped; follow-up angiography at 12 months still demonstrated minimal residual filling of both aneurysms. The authors speculated that PEDs within the thrombosed portion of these fusiform aneurysms may endothelialize at a rate far slower than that observed when a similarly sized PED was placed across a non-fusiform aneurysm. Similar results were observed in our study, in which the mean time to complete occlusion of fusiform aneurysms confirmed by first angiographic exceeded 12 months, which was significantly longer than the time to occlusion for saccular aneurysms. The four patients with fusiform aneurysms who experienced in-stent thrombosis in our study showed residual aneurysm filling at the final angiographic follow-up. Antiplatelet agents were discontinued in these patients because of gastrointestinal bleeding or planned surgery. Therefore, discontinuation of antiplatelet therapy may never be safe after flow diversion in patients with large or giant BA fusiform aneurysms with residual filling. When persistent residual filling is encountered months after PED reconstruction, Klisch et al. (33) suggested that placement of additional devices is preferable to altering the antiplatelet regimen.

Delayed aneurysmal rupture is another potentially serious complication of FD treatment that can have a devastating outcome. Hou et al. (34) performed a systematic review of patients who experienced delayed rupture after FD placement and concluded that increased intra-aneurysmal pressure, destabilization of the aneurysm wall by intra-aneurysmal thrombus, persistent residual intra-aneurysmal flow, large or giant size, presence of symptoms, and FD-induced mechanical injury might contribute to delayed rupture. In our series, four patients with aneurysm sizes ranging from 22 to 40 mm developed delayed rupture; three occurred from aneurysms located in the proximal segment, including two with a VBJ aneurysm that was treated with a single PED without coiling and contralateral vertebral sacrifice. For VBJ aneurysms treated using the PED, coil occlusion of the contralateral vertebral artery is required to prevent disease progression (35). Coiling can also provide protection from hemorrhagic complications by changing intra-aneurysmal flow dynamics and controlling intra-aneurysmal thrombosis (17); however, coiling may worsen mass effect in some cases (36). Our pooled analysis demonstrated a 4.6% rate (range, 0–25) of worsening mass effect after FD treatment of BA aneurysms (Table 4). Worsening of mass effect after treatment may be associated with aneurysm thrombosis, increase in maximal aneurysm diameter, and new adjacent edema (37). Several studies (12, 35, 38) have shown that early management before compressive symptoms develop is important to achieve a good clinical outcome. In our study, three patients who experienced mass effect symptoms postoperatively had symptoms of brainstem compression before PED treatment. Two of these patients had intermittent episodic symptoms for less than 2 months before treatment and ultimately had a favorable clinical outcome. Another patient had severe brainstem compression symptoms for 6 months prior to treatment and experienced worsened mass effect after treatment with a single PED and adjunctive coiling. She eventually died of mass effect-related brainstem failure. Although initial clinical presentation was not associated with complications or poor outcome in our study, we believe that early management of patients with symptomatic mass effect can achieve favorable clinical outcomes.

## Limitations

Our study is limited by its single-center retrospective design and relatively small sample size, which may have introduced statistical bias.

## Conclusion

The results of this small series suggest that PED treatment of large or giant BA aneurysms is effective and can achieve a satisfactory long-term occlusion rate. However, the treatment complications are not negligible. Aneurysm size is the strongest predictor of perioperative complications and poor clinical outcome.

## Data Availability Statement

The original contributions presented in the study are included in the article/supplementary material, further inquiries can be directed to the corresponding authors.

## Ethics Statement

The studies involving human participants were reviewed and approved by the Ethics Committee of Beijing Tiantan Hospital. Written informed consent to participate in this study was provided by the participants' legal guardian/next of kin.

## Author Contributions

YL and ML: conception and design. KL, LZ, YZ, YJ, PL, and HH: analysis and interpretation of data. XC and HG: drafting the article. YL: approved the final version of the manuscript on behalf of all authors. All authors critically revised the article. All authors contributed to the article and approved the submitted version.

## Funding

This study was supported by the National Key Research and Development Program of China (grant no. 2017YFB1304400) and Youth Program of National Natural Science Foundation of China (grant no. 81901197).

## Conflict of Interest

The authors declare that the research was conducted in the absence of any commercial or financial relationships that could be construed as a potential conflict of interest.

## Publisher's Note

All claims expressed in this article are solely those of the authors and do not necessarily represent those of their affiliated organizations, or those of the publisher, the editors and the reviewers. Any product that may be evaluated in this article, or claim that may be made by its manufacturer, is not guaranteed or endorsed by the publisher.
